# Why Seizures Break Through: Distinguishing Background Susceptibility From Proximal Precipitants in Patients With Epilepsy

**DOI:** 10.7759/cureus.116904

**Published:** 2026-09-25

**Authors:** Mahdi Fadel, Ali Fadel, Nataliah R Jwaida, Burton Tabaac

**Affiliations:** 1 Medicine, American University of the Caribbean School of Medicine, Cupecoy, SXM; 2 Medicine, Wayne State University, Detroit, USA; 3 Neurology, University of Nevada, Reno, USA

**Keywords:** antiseizure medication, breakthrough seizure, epilepsy, seizure activity, seizure triggers

## Abstract

Breakthrough seizures are commonly described as unprovoked seizures that recur in patients with epilepsy who remain on antiseizure medication (ASM) after a sustained period of seizure freedom. These events are clinically important because they can disrupt previously stable seizure control and alter counseling, safety considerations, and treatment decisions. The literature often combines two conceptually different explanations: background factors that reflect a greater underlying propensity for recurrence and proximal exposures that may help explain why a seizure occurs at a particular time. This focused narrative review distinguishes these concepts and evaluates the strength of evidence supporting each. Breakthrough-specific studies suggest that greater prior seizure burden, difficulty achieving remission, neurological impairment, treatment history, and epilepsy etiology may reflect greater background susceptibility. In contrast, frequently cited precipitants such as medication nonadherence, sleep deprivation, psychological stress, infection, alcohol exposure, and pregnancy vary considerably in evidentiary strength. Nonadherence has relatively strong mechanistic and objective support, although inadequate medication exposure can complicate whether an event should be classified as a breakthrough seizure. Sleep deprivation and stress are commonly reported and biologically plausible, but their independent causal contributions remain uncertain. Heavy alcohol exposure appears more clinically relevant than modest intake. Pregnancy may alter ASM exposure in a medication-specific manner, but available evidence does not support treating pregnancy as a uniform independent cause of seizure worsening. Precipitants may also cluster, making attribution to a single exposure potentially misleading. Broader electrophysiological evidence further suggests that underlying seizure susceptibility may fluctuate over time. Distinguishing background susceptibility from proximal precipitants provides a practical framework for prognosis, counseling, medication management, and future research while emphasizing the difference between association and demonstrated causation.

## Introduction and background

Breakthrough seizures represent an important but comparatively understudied problem in epilepsy care. Although no internationally accepted definition exists, the term is generally used to describe an unprovoked seizure occurring in a patient with epilepsy who remains on antiseizure medication (ASM) after at least 12 months of seizure freedom [1,2]. The clinical importance of such events extends beyond the seizure itself. Because they occur after a prolonged period of control, breakthrough seizures may be unexpected, expose patients to injury, reinstate driving restrictions, disrupt employment and personal independence, and prompt substantial changes in treatment [1,2].

Breakthrough seizures are also not rare. In the Standard versus New Antiepileptic Drugs (SANAD) analysis, 536 of 1,593 patients who had achieved 12-month remission subsequently experienced a first breakthrough seizure, corresponding to an estimated two-year risk of 37% [2]. More recently, Doerrfuss et al. observed breakthrough seizures in 29% of 521 patients during longitudinal follow-up after at least 12 months of seizure freedom while taking ASM [1]. Unlike the SANAD value, this represents an observed cohort proportion rather than a fixed two-year cumulative risk estimate. These findings are clinically important because a breakthrough event may not be isolated, and some patients experience further seizures or difficulty re-establishing sustained seizure freedom [1,2].

Understanding why seizures break through is therefore relevant at several clinical decision points. Before a breakthrough seizure occurs, clinicians counsel seizure-free patients regarding prognosis and whether continued ASM treatment remains necessary. After an event, the clinical question changes: did the seizure reflect a more refractory underlying epilepsy, a temporary and potentially preventable exposure, inadequate medication exposure, or some interaction among these factors?

Answering these questions requires separating two concepts that are frequently grouped together in discussions of seizure recurrence. Background risk factors identify an increased underlying propensity to experience breakthrough seizures over the disease course but do not directly precipitate the individual event. Proximal precipitants, in contrast, are transient behavioral, physiological, pharmacological, or environmental exposures that may contribute to why a seizure occurs at a particular time [1].

This distinction is clinically useful because the two categories imply different actions. Background susceptibility informs prognosis and may contribute to counseling regarding long-term therapy. Proximal precipitants are potentially actionable and therefore form the basis of recommendations regarding medication adherence, sleep, alcohol exposure, stress, illness, and other behaviors. The distinction also demands different standards of evidence. 

Previous reviews have examined provocative factors, reflex seizures, spontaneous seizure worsening, and external precipitants, particularly in general epilepsy and drug-resistant epilepsy populations [3-5]. However, these literature studies have not been organized specifically around the distinction between background susceptibility and proximal precipitants in patients who have already achieved sustained seizure freedom. This focused narrative review applies that framework to breakthrough seizures, evaluates the strength and directness of evidence on both sides, examines where the distinction becomes blurred, and identifies implications for clinical practice and future research. For the purposes of this review, breakthrough seizures are operationally considered seizures occurring after at least 12 months of seizure freedom while patients remain on ASM, consistent with the definitions used in the principal breakthrough-specific cohorts [1,2].

## Review

Methodology

Review Design

This study was conducted as a focused narrative review examining background risk factors and proximal precipitants associated with breakthrough seizures in patients with epilepsy. A narrative approach was selected because the available literature is limited and heterogeneous, encompassing prognostic cohort studies specifically examining breakthrough seizures, prospective observational studies of seizure precipitants, self-report studies, systematic and narrative reviews, pharmacokinetic investigations, and studies of endogenous seizure rhythmicity. The principal organizing framework was the distinction between background susceptibility and proximal precipitants, with particular emphasis on whether evidence was derived directly from patients who had achieved sustained seizure freedom or extrapolated from broader populations with epilepsy.

Data Sources

Relevant literature was identified through searches of PubMed/MEDLINE and Google Scholar, supplemented by OpenEvidence, an AI-assisted medical literature retrieval tool, and manual review of the reference lists of pertinent publications. The final literature search was conducted on September 20, 2026. OpenEvidence was used only to identify potentially relevant publications; study characteristics, numerical results, and claims incorporated into the review were verified against the original peer-reviewed publications before citation.

Search Terms

Searches combined terms related to breakthrough seizures with terms describing recurrence, prognostic factors, and proposed precipitants. The representative PubMed query used to generate the update-search count was: (“breakthrough seizure”[tiab] OR “breakthrough seizures”[tiab]) AND (epilepsy[tiab] OR “antiseizure medication”[tiab] OR anticonvulsant[tiab]). Additional targeted searches combined “breakthrough seizure,” “seizure recurrence,” “seizure remission,” or “seizure freedom” with “acute symptomatic seizure,” “antiseizure medication withdrawal,” “nonadherence,” “drug interaction,” “proconvulsant medication,” “infection,” “pregnancy pharmacokinetics,” “seizure cycles,” “circadian,” and “multidien.” No lower publication-date limit was imposed. No formal publication-type filter was applied.

Source Selection

Studies were selected according to their relevance to the review question and the directness of their evidence to the operational breakthrough-seizure population. Priority was given to studies defining breakthrough seizures after sustained seizure freedom while patients remained on ASM. Original clinical studies examining breakthrough-seizure risk, prognosis, or potential precipitants were prioritized. Systematic and narrative reviews, professional guidance, pharmacokinetic studies, and studies from broader epilepsy populations were included when they provided evidence relevant to a question for which breakthrough-specific data were limited. Evidence from general epilepsy populations, uncontrolled epilepsy, acute seizure admissions, or drug-resistant epilepsy was considered supportive or indirect rather than equivalent to breakthrough-specific evidence. Titles and abstracts were reviewed for relevance, followed by full-text review of potentially relevant publications. No formal language filter was applied during searching; however, all publications ultimately included in the synthesis were available in English. The final PubMed update search on September 20, 2026 identified 215 records. After title and abstract review, 52 publications were assessed in full text. Three additional relevant studies identified during the revision process were incorporated into the final synthesis, including evidence addressing alternative breakthrough-seizure definitions and pregnancy-related seizure frequency. A total of 24 publications were included in the final synthesis. Google Scholar, OpenEvidence, and reference-list review were used as supplementary targeted retrieval methods rather than as sources for a formal PRISMA-style screening count. 

Evidence Appraisal

Evidence was qualitatively assessed according to study design, sample size, directness to the breakthrough-seizure population, objective versus self-reported exposure measurement, presence of comparison periods or control groups, potential confounding, and the ability of the study design to establish temporality or causality. For proposed precipitants, particular attention was given to recall bias, ascertainment bias, co-occurrence of multiple precipitants, and whether candidate exposures were measured during seizure-free comparison periods as well as before seizures. Any descriptions of evidence strength used in this review are qualitative and do not represent a formal validated evidence-grading or risk-of-bias framework.

Data Synthesis

Findings were synthesized narratively and organized according to background susceptibility, proximal precipitants, interactions among precipitants, endogenous fluctuations in seizure susceptibility, clinical implications, and limitations of causal inference. Breakthrough-specific evidence was emphasized whenever available, while evidence derived from broader epilepsy populations was identified as indirect or supportive.

Limitations of the Review Methodology

Because this review was narrative in scope, PRISMA methodology, formal risk-of-bias scoring, quantitative synthesis, and meta-analysis were not performed. The available literature spans heterogeneous study designs, patient populations, seizure-control states, and methods of identifying potential precipitants, limiting direct comparison among studies. An additional limitation is the absence of an internationally standardized definition of breakthrough seizures. Furthermore, much of the literature regarding proposed precipitants was conducted in general or uncontrolled epilepsy populations rather than specifically in patients who had maintained seizure freedom for at least 12 months. The review therefore aimed to distinguish direct breakthrough-specific evidence from supporting evidence, critically assess the strength of reported associations, and identify limitations and gaps in the current evidence base.

Defining breakthrough seizures

A standardized definition of breakthrough seizure has not been established. SANAD defined a breakthrough seizure as one occurring after at least 12 months of remission while the patient remained on treatment [2]. Doerrfuss et al. similarly defined a breakthrough seizure as an unprovoked seizure occurring while taking ASM after at least 12 months of seizure freedom, but additionally excluded patients who had undergone ASM alteration or dose reduction during the preceding three months [1]. For the purposes of this review, a “precipitant” refers to a temporally proximal exposure that may contribute to seizure occurrence without implying that the event meets criteria for an acute symptomatic (provoked) seizure. This distinction is important because seizures occurring in sufficiently close temporal association with severe metabolic derangements, drug or alcohol intoxication or withdrawal, or acute central nervous system insults may instead meet criteria for acute symptomatic seizures and represent a distinct nosologic category [6]. Similarly, seizure recurrence following planned ASM withdrawal represents a related but distinct recurrence paradigm and is not considered a breakthrough seizure on unchanged therapy in this review [7]. The principal distinctions between the operational definition of breakthrough seizure used in this review and acute symptomatic seizures are summarized in Table 1.

**Table 1:** Distinguishing breakthrough seizures from acute symptomatic seizures ASM: antiseizure medication; CNS: central nervous system; SANAD: Standard versus New Antiepileptic Drugs. Breakthrough seizure follows the operational definition used by SANAD and the study by Doerrfuss et al. [1,2]. Acute symptomatic seizure classification follows the study by Beghi et al. [6]. Recurrence after planned ASM withdrawal is considered separately [7].

Feature	Breakthrough seizure in this review	Acute symptomatic seizure
Definition	An unprovoked seizure occurring in a patient with established epilepsy after ≥12 months of seizure freedom while remaining on ASM [1,2].	A seizure occurring in close temporal association with an acute systemic or central nervous system insult [6].
Relationship to prior seizure control	Requires a preceding period of sustained seizure freedom under the operational definition used in this review [1,2].	Prior seizure freedom is not required; classification depends on the temporal relationship to an acute provoking condition [6].
Relationship to ASM therapy	Occurs while the patient remains on ASM. Nonadherence, recent dose reduction, or inadequate drug exposure may complicate whether an event represents a true breakthrough seizure [1]. Planned ASM withdrawal is treated separately in this review [7].	Classification is based primarily on the presence of an acute provoking insult rather than failure of chronic ASM therapy [6].
Examples or clinical contexts	Recurrence after sustained seizure control in the absence of an acute condition sufficient to classify the event as acute symptomatic.	Acute CNS insult or infection, severe metabolic derangement, qualifying drug or alcohol intoxication or withdrawal, or exposure to an epileptogenic drug [6].
Interpretation	Prompts evaluation of background epilepsy susceptibility, adequacy of ASM exposure, and potentially reversible proximal factors.	Represents a distinct nosologic category in which evaluation and management focus on the acute provoking condition.

The 12-month threshold is clinically convenient but represents a convention rather than a biologically established boundary. The 12-month threshold was selected because it was used by SANAD and Doerrfuss et al., the two principal adult cohorts providing breakthrough-specific prognostic evidence [1,2]. Other studies have used broader definitions. Aldosari et al. required only two months of preceding seizure freedom in children, and Prasad and Verma included patients who had received antiseizure therapy for at least six months without requiring a 12-month remission interval [8,9]. These differences may increase reported event rates and change the mix of precipitants by including patients with shorter or less firmly established control. Such studies are therefore treated as supportive rather than directly equivalent. A different threshold could change prevalence estimates and cohort composition, but it would not change the distinction between background susceptibility and proximal precipitants. In the Doerrfuss cohort, the median seizure-free interval preceding a breakthrough seizure was 34 months, demonstrating that many events occur well beyond the minimum qualifying period [1].

A more difficult definitional issue is medication nonadherence. Breakthrough seizures are generally interpreted as evidence that previously effective treatment no longer provides complete control. However, a seizure occurring because treatment was not taken as prescribed may instead reflect inadequate medication exposure rather than failure of the underlying therapeutic regimen. A seizure attributed to medication nonadherence may not necessarily indicate inadequate seizure control [1]. Future definitions should therefore specify not only the qualifying seizure-free interval but also how medication nonadherence, deliberate withdrawal, and recent dose alterations are classified.

Background susceptibility factors

Seizure Burden Before Remission

In SANAD, the number of tonic-clonic seizures accumulated before achievement of 12-month remission was independently associated with subsequent breakthrough risk [2]. Greater seizure burden before control may therefore provide information regarding the underlying propensity of an individual's epilepsy to re-emerge despite treatment.

The prognostic effects became clinically substantial when risk factors were considered together. Patients without neurological insult, with only one previous tonic-clonic seizure, who achieved remission promptly had an estimated 31% risk of breakthrough seizure within two years. At the opposite extreme, patients with neurological insult, approximately 20 prior tonic-clonic seizures, and three years required to achieve remission had an estimated two-year risk of 71% [2].

Time to Remission

Time required to reach sustained seizure freedom was also an independent predictor of breakthrough seizure in SANAD [2]. This finding supports the interpretation that rapid response to treatment may mark a more treatment-responsive disease course, whereas prolonged difficulty reaching remission reflects greater background susceptibility.

Time-related variables may also provide prognostic information after a breakthrough seizure. In the Doerrfuss cohort, patients who failed to re-achieve terminal 12-month seizure freedom had experienced a significantly shorter period of seizure freedom before the breakthrough event than those who subsequently regained control [1].

Neurological Impairment and Intellectual Disability

Neurological impairment and intellectual disability have both been associated with increased breakthrough seizure risk. Neurological insult, defined by learning difficulty or functionally significant neurological deficit, was identified as an independent predictor [2]. Intellectual disability was similarly associated with breakthrough seizures (adjusted OR 2.768, 95% CI 1.271-6.031) [1]. Together, these findings suggest that underlying neurological impairment may indicate greater difficulty maintaining long-term seizure control. However, intellectual disability was identified from existing clinical documentation rather than a standardized cognitive assessment, which may limit classification precision [1].

Treatment history and ASM burden

A greater number of previously and currently tried ASMs were independently associated with breakthrough seizures, with an adjusted OR of 1.203 per additional ASM (95% CI 1.056-1.371) [1]. This is more likely to represent a history of greater treatment difficulty than a direct effect of ASM exposure.

Patients with breakthrough seizures also had a higher median current drug load, although this variable was excluded from the principal risk-factor model because of potential retrospective bias. Drug load can change during follow-up, particularly as patients with longer seizure-free periods have more opportunity for medication reduction, making it less suitable as a stable baseline risk factor [1].

Epilepsy type and etiology

Breakthrough seizure risk also varied according to epilepsy etiology [1]. Post-ischemic-stroke epilepsy was associated with lower odds of breakthrough seizure (adjusted OR 0.267, 95% CI 0.075-0.946), as was genetic generalized epilepsy (adjusted OR 0.559, 95% CI 0.319-0.978). In contrast, crude breakthrough rates were 47.8% among patients with traumatic brain injury-related epilepsy and 43.5% among those with hippocampal sclerosis. Neither, however, remained significantly associated after multivariable adjustment (adjusted OR 1.900, 95% CI 0.772-4.676 and adjusted OR 1.268, 95% CI 0.504-3.193, respectively) [1].

These findings should be interpreted cautiously because some etiologic subgroups were small and produced wide confidence intervals. Nevertheless, they suggest that the underlying disease substrate may continue to influence seizure susceptibility even after prolonged seizure freedom. The principal background susceptibility factors and limitations of the supporting evidence are summarized in Table 2.

**Table 2:** Evidence summary of background factors associated with breakthrough seizures ASM: antiseizure medication; CI: confidence interval; OR: odds ratio; SANAD: Standard versus New Antiepileptic Drugs; TBI: traumatic brain injury.

Background factor	Main supporting study	Study design/population	Key finding	Interpretation	Evidence limitations
Number of tonic-clonic seizures before remission	Bonnett et al. (SANAD) [2]	Analysis of 1,593 SANAD participants who achieved 12-month remission	A greater number of tonic-clonic seizures before remission independently predicted subsequent breakthrough seizure.	Higher pre-remission seizure burden may identify greater baseline epilepsy susceptibility.	Prognostic association; it does not establish a causal mechanism.
Time required to achieve 12-month remission	Bonnett et al. (SANAD) [2]	SANAD prognostic analysis	Longer time to remission independently predicted breakthrough seizure.	Delayed seizure control may mark less treatment-responsive epilepsy and greater background susceptibility.	May overlap with other markers of disease severity and treatment difficulty.
Neurological insult	Bonnett et al. (SANAD) [2]	SANAD prognostic analysis	Neurological insult was independently associated with breakthrough seizure.	Underlying neurological injury may carry prognostic information beyond seizure type alone.	The study definition included learning difficulty or a functionally significant neurological deficit.
Intellectual disability	Doerrfuss et al. [1]	Retrospective tertiary epilepsy outpatient cohort; 521 patients seizure-free for at least 12 months while taking ASM	Intellectual disability was independently associated with breakthrough seizure (adjusted OR 2.768, 95% CI 1.271-6.031).	May identify greater baseline vulnerability and a more difficult-to-control epilepsy course.	“Known intellectual disabilities” were retrieved from the electronic database, so ascertainment depended on existing clinical documentation.
Historical ASM burden	Doerrfuss et al. [1]	Retrospective tertiary epilepsy outpatient cohort	The number of ASMs previously and currently tried was independently associated with breakthrough seizure (adjusted OR 1.203 per additional ASM, 95% CI 1.056-1.371).	More prior ASM exposure is better interpreted as a marker of difficulty achieving seizure control than as a causal exposure.	Current drug load was excluded from the principal risk-factor model because retrospective follow-up could bias this dynamic treatment variable.
Epilepsy type and etiology	Doerrfuss et al. [1]	Retrospective tertiary epilepsy outpatient cohort	Post-ischemic stroke epilepsy (adjusted OR 0.267, 95% CI 0.075-0.946) and genetic generalized epilepsy (adjusted OR 0.559, 95% CI 0.319-0.978) had lower adjusted odds. Crude breakthrough rates were 47.8% for TBI and 43.5% for hippocampal sclerosis.	The underlying epilepsy substrate may continue to influence recurrence susceptibility after prolonged seizure freedom.	Some etiologic subgroups were small. TBI and hippocampal sclerosis rates were crude; neither was independently significant after multivariable adjustment.

Clinical course after a breakthrough seizure

A breakthrough seizure may mark a clinically important transition from sustained seizure control to a period of increased recurrence risk. In SANAD, the estimated risk of another seizure within two years after a first breakthrough event was 74%, with recurrence occurring relatively early among those affected; the median time to the next seizure was approximately 31 days [2]. Recurrence was not always limited to a single additional event, as 45% experienced more than one seizure before subsequently achieving another 12-month remission period [2].

Recovery of sustained seizure control is also not universal. Among 108 patients with at least 12 months of post-breakthrough follow-up, the median follow-up duration was 39 months (interquartile range: 29-71 months), and 37 patients (34.3%) had not re-achieved terminal 12-month seizure freedom by their last visit [1]. Thus, the post-breakthrough course appears heterogeneous: some patients regain prolonged seizure control, whereas others experience early recurrence, repeated seizures, or persistent difficulty re-establishing remission. This variability highlights the importance of determining whether a breakthrough event reflects greater underlying epilepsy susceptibility, a reversible proximal precipitant, or an interaction between both.

Proximal precipitants of seizures

Patients with epilepsy commonly report circumstances that appear to precede seizure occurrence. In a study of 1,677 individuals with epilepsy, more than half reported at least one perceived precipitant, most often emotional stress, sleep deprivation, or tiredness [3]. Broader literature on provocative and reflex seizures similarly implicates transient physiological, behavioral, and environmental exposures, including stress, fatigue, fever, sleep loss, menstrual changes, heavy alcohol exposure, and visual stimulation [4].

The relevance of individual precipitants may also vary across epilepsy syndromes. In a survey of 400 patients, Frucht et al. found that 62% reported at least one precipitant, with stress, sleep deprivation, illness, and fatigue among the most frequently identified exposures; the ranking of precipitants also differed among epilepsy syndromes [10]. Stress, fatigue, and sleep deprivation were positively correlated, further suggesting that perceived precipitants may cluster rather than act independently. However, these findings were based on patient-reported exposures in a general epilepsy population and were not restricted to breakthrough seizures after sustained remission, limiting direct application to the population considered in this review [10].

More recent evidence further illustrates both the relevance of proposed precipitants and the importance of definitional differences. A 2025 study of 300 patients with epilepsy reported breakthrough seizures in 46%, with significant associations involving medication nonadherence, number of antiseizure medications, treatment duration, awareness of triggering factors, and sleep deprivation; stress was not significantly associated [9]. Eligibility required at least six months of seizure freedom while receiving antiseizure therapy rather than the ≥12-month seizure-free interval used in this review. Those findings therefore provide recent supportive evidence but are not directly comparable with SANAD or Doerrfuss et al. [1,2].

Whether these observations translate directly to breakthrough seizures after sustained remission is less certain. In the breakthrough-specific cohort, medication nonadherence was the most frequently identified presumed precipitant, occurring in 10.6% of cases, followed by sleep deprivation and acute infection at 5.3% each, excessive stress at 4.6%, and pregnancy at 3.3% [1]. However, identifying a precipitant was often not possible: 35.1% had no presumed trigger despite documented inquiry, while another 33.1% lacked adequate trigger documentation [1].

Taken together, these findings suggest that proximal precipitants may contribute to some breakthrough seizures, but they do not provide a complete explanation for most events. The high proportion of cases without an identifiable or adequately documented trigger also highlights an important limitation of the current evidence and reinforces the need to distinguish patient-reported temporal associations from established causal precipitants.

Antiseizure medication nonadherence

Medication nonadherence has one of the clearest mechanistic links to seizure recurrence because missed doses reduce therapeutic drug exposure. It was also the most frequently documented presumed precipitant in the breakthrough-specific cohort [1].

Broader evidence supports its clinical importance. In a prospective study of 282 patients admitted with seizures, therapeutic drug monitoring identified nonadherence in 39% of admissions, including 24% classified as definite and 15% as probable [11]. Notably, 44% of patients objectively classified as nonadherent nevertheless reported taking their medication regularly, highlighting the limitations of relying on self-reported adherence [11]. Because this cohort included acute seizure admissions rather than exclusively patients with breakthrough seizures after prolonged remission, these findings support the relevance of nonadherence without establishing how often it directly causes narrowly defined breakthrough events. Nonadherence therefore represents both a proximal precipitant and a potential source of misclassification when defining true treatment failure. 

A further limitation is that current breakthrough-specific evidence does not consistently distinguish recurrence despite objectively confirmed therapeutic ASM exposure from seizures associated with nonadherence or subtherapeutic exposure. In the study by Doerrfuss et al., presumed triggers, including nonadherence, were retrospectively identified from clinical documentation based on treating-physician attribution rather than systematic therapeutic drug monitoring [1]. By contrast, Samsonsen et al. used therapeutic drug monitoring to identify nonadherence, but that study examined acute seizure admissions rather than patients meeting the ≥12-month operational definition used in this review [11]. The available literature therefore does not permit a reliable estimate of how often narrowly defined breakthrough seizures represent pharmacological treatment failure despite adequate ASM exposure versus recurrence associated with insufficient exposure.

Reduced effective ASM exposure and medication-related factors

Transient loss of seizure control may also occur despite reported adherence. Bauer and Quigg describe unanticipated changes in ongoing medical therapy, concurrent medications that alter ASM pharmacokinetics, and inadvertent exposure to proconvulsant medications as potential contributors to transient breakthrough seizures in medically responsive epilepsy [12]. This broadens the medication-related framework beyond missed doses: some events may reflect reduced pharmacologic protection or a lowered seizure threshold rather than increased intrinsic refractoriness. However, this evidence derives primarily from a clinical review of medically responsive epilepsy rather than a prospective cohort meeting the narrower breakthrough definition used in this review and should therefore be regarded as supportive rather than breakthrough-specific evidence. Similarly, a seizure occurring in sufficiently close temporal association with exposure to a recognized epileptogenic medication may meet criteria for an acute symptomatic seizure rather than the operational breakthrough-seizure definition used here [6].

Sleep deprivation

Sleep deprivation is widely regarded as a seizure trigger and is commonly addressed during counseling and used to increase the diagnostic yield of electroencephalography. However, evidence that insufficient sleep directly precipitates spontaneous clinical seizures is less certain than its widespread acceptance suggests.

A systematic re-evaluation found that although sleep deprivation can increase interictal epileptiform discharges, studies examining its longitudinal relationship with clinical seizure risk were generally low quality and produced conflicting results, preventing firm conclusions about causality [13]. In contrast, a prospective case-crossover study of 179 seizure admissions found shorter sleep duration before seizure events than during seizure-free follow-up periods, supporting an association between shorter sleep duration and seizure occurrence in that population [14]. However, sleep duration also interacted substantially with alcohol and other exposures.

Overall, sleep deprivation remains biologically plausible and is supported by some prospective evidence, but current studies do not establish that sleep loss independently causes breakthrough seizures after sustained remission.

Psychological stress

Psychological stress is among the most frequently reported seizure precipitants, but its role is difficult to isolate. Unlike medication exposure or sleep duration, stress is subjective and often overlaps with anxiety, depression, sleep disruption, alcohol use, and medication adherence.

Prospective data suggest that stress-related vulnerability may influence seizure recurrence. In adults with a first unprovoked seizure or newly diagnosed epilepsy, lifetime generalized anxiety disorder was associated with an approximately threefold greater hazard of recurrence, while low neighborhood collective efficacy was associated with an approximately 2.7-fold greater hazard; lifetime mood disorder was also associated with recurrence in an alternate model [15]. Because these were lifetime or contextual characteristics rather than acute pre-seizure measurements, they are better interpreted as markers of background vulnerability than as evidence that an episode of acute stress directly precipitated a seizure.

A 2025 systematic review of 34 studies similarly found that perceived stress was identified as a seizure precipitant in 85% of included studies, but substantial differences in stress measurement, patient populations, seizure control, and confounding limited interpretation [16]. Longitudinal findings were also inconsistent: one diary-based study linked greater perceived stress with increased next-day seizure likelihood, whereas another found no significant association [16].

A focused review by McKee and Privitera similarly identified stress as a commonly reported seizure precipitant and noted greater anxiety and depression among patients who reported stress-related seizures [17]. Although small prospective studies of stress-reduction interventions have shown benefits in some outcomes, effects on seizure frequency have been inconsistent [17]. Taken together with the newer systematic evidence, these findings reinforce the distinction between frequent patient attribution of stress and demonstration of an independent causal effect on seizure occurrence.

Overall, stress appears clinically relevant to seizure activity, but current evidence more strongly supports an association with seizure susceptibility than a simple, independent causal relationship with breakthrough seizures after sustained remission.

Infection and systemic illness

Acute infection was documented as a presumed precipitant in 5.3% of breakthrough seizures [1]. However, because potential triggers were assessed only after breakthrough events had occurred, these data establish an association rather than an independent causal effect. Importantly, a seizure occurring with an acute CNS infection or a sufficiently severe systemic or metabolic disturbance meeting accepted acute symptomatic seizure criteria should be classified separately rather than as a breakthrough seizure under the operational framework used in this review [6].

Acute illness is particularly difficult to isolate because infection often occurs alongside fever, sleep disruption, dehydration, reduced oral intake, vomiting, metabolic abnormalities, altered medication absorption, or missed ASM doses. A seizure during infection may therefore reflect the combined influence of several transient factors rather than infection alone. Prospective studies comparing these exposures during seizure and seizure-free periods are needed to determine their independent contributions.

Alcohol

The relationship between alcohol and seizure recurrence appears to depend strongly on the amount consumed and the circumstances surrounding exposure. In a cohort of 310 adults with epilepsy, 18.1% of those who consumed alcohol reported alcohol-related worsening of seizures [18]. These events followed relatively heavy intake, with all affected patients consuming at least seven standard drinks, and 95% of reported alcohol-related seizures occurring within 12 hours after cessation of drinking. Genetic generalized epilepsy and chronic heavier alcohol use were independently associated with greater risk [18].

Prospective crossover data further suggest that modest alcohol consumption should be distinguished from hazardous exposure. Among patients who drank socially without hazardous use or excessive binge drinking, alcohol intake before seizures did not differ from intake during seizure-free periods [19]. In contrast, binge drinking and withdrawal were associated with loss of seizure control, while sleep loss frequently accompanied alcohol exposure [19]. Seizures occurring in the setting of qualifying alcohol withdrawal or intoxication may meet criteria for acute symptomatic seizures and therefore should not automatically be classified as breakthrough events [6].

Taken together, these findings argue against treating alcohol as a uniform seizure precipitant. Heavy or binge consumption, withdrawal, and accompanying factors may be more clinically relevant than modest intake alone. Alcohol-related breakthrough seizures may therefore reflect a cascade of interacting exposures rather than the independent effect of alcohol itself.

Pregnancy and altered ASM exposure

Pregnancy was identified as a presumed precipitant in 3.3% of breakthrough seizures in the Doerrfuss cohort [1], but this retrospective attribution does not establish that pregnancy itself increases breakthrough-seizure risk. One plausible pathway is pregnancy-related alteration in ASM pharmacokinetics, which may reduce effective medication exposure in a drug-specific manner.

The prospective Maternal Outcomes and Neurodevelopmental Effects of Antiepileptic Drugs (MONEAD) study demonstrated substantial changes in dose-normalized ASM concentrations during pregnancy compared with postpartum levels, including reductions of up to 56.1% for lamotrigine, 36.8% for levetiracetam, 32.6% for oxcarbazepine, 39.9% for lacosamide, and 29.8% for zonisamide [20]. The magnitude of pharmacokinetic change therefore varies among individual ASMs. Importantly, these concentration changes establish altered pharmacokinetics rather than demonstrating that pregnancy itself causes breakthrough seizures.

Clinical seizure-frequency data provide additional context. In a prospective MONEAD analysis, seizure frequency increased during the pregnancy/peripartum epoch relative to the postpartum epoch in 70 of 299 pregnant women (23%) compared with 23 of 93 nonpregnant controls (25%; OR 0.93, 95% CI 0.54-1.60) [21]. ASM dose changes were substantially more frequent during pregnancy [21]. These findings do not support pregnancy as a uniform independent precipitant of seizure worsening; rather, pregnancy may create medication-specific changes in effective exposure that warrant clinical monitoring and individualized dose adjustment. Neither MONEAD analysis was restricted to patients meeting the ≥12-month operational breakthrough-seizure definition used in this review, so its application to narrowly defined breakthrough seizures remains indirect [20,21].

Menstrual cycling and other hormonal influences have also been reported to modify seizure occurrence in the broader epilepsy literature [4]. However, current evidence is not sufficiently specific to breakthrough seizures after sustained remission to support firm causal conclusions.

Clustering and the cascade of precipitants

The assumption that a seizure can be attributed to a single identifiable precipitant is often unrealistic. In a prospective study of 152 acute seizure admissions, multiple potential precipitants were assessed simultaneously using sleep diaries, therapeutic drug monitoring, alcohol screening, and standardized measures of anxiety, depression, and patient-perceived triggers [22].

Considerable overlap was found among these exposures. Sleep loss was associated with hazardous drinking and anxiety, while perceived stress correlated with both anxiety and depression. Patients also did not always accurately identify the factors most relevant to their seizures: those with objectively demonstrated medication nonadherence often attributed little importance to missed doses, and patients with harmful alcohol use similarly tended to underestimate alcohol as a contributor [22].

These findings support a cascade model in which several transient exposures may interact rather than act independently. Stress may disrupt sleep, alcohol use may further impair sleep or medication adherence, and these changes may coincide with periods of heightened underlying seizure susceptibility. Attributing the resulting seizure to any single factor may therefore oversimplify the clinical course.

Endogenous seizure rhythms and dynamic susceptibility

The distinction between background risk factors and proximal precipitants assumes that underlying seizure susceptibility is relatively stable. Long-term electrophysiological studies, however, suggest that susceptibility may fluctuate over time.

In 222 adults with medically refractory focal epilepsy, circadian seizure cycles were identified in 89% of evaluable patients, multidien cycles in 60%, and circannual cycles in 12%, with seizures clustering during specific phases of these rhythms [23]. Long-term implanted-device recordings have similarly demonstrated stable, patient-specific circadian and multidien patterns of epileptiform activity, with seizures occurring preferentially during particular phases of multidien cycles [24].

These populations differ substantially from patients with well-controlled epilepsy, so the findings cannot be directly extrapolated to breakthrough seizures. Nevertheless, they suggest that seizure susceptibility may vary even in the absence of an identifiable external exposure.

This supports a more dynamic model in which breakthrough seizures may result from the interaction of baseline epilepsy susceptibility, a transient period of heightened endogenous risk, and a proximal precipitant. Under this framework, an exposure such as sleep loss, stress, illness, or alcohol may have little effect during a lower-risk period but contribute to seizure occurrence when underlying susceptibility is temporarily increased. Evidence for the major proposed proximal precipitants and their interactions is summarized in Table 3.

**Table 3:** Evidence summary of major proposed proximal precipitants and their interactions ASM: antiseizure medication; MONEAD: Maternal Outcomes and Neurodevelopmental Effects of Antiepileptic Drugs.

Proposed precipitant	Main supporting evidence	Key finding	Interpretation	Major limitations
ASM nonadherence	Doerrfuss et al. [1]; Samsonsen et al. [11]	Nonadherence was documented as a presumed precipitant in 10.6% of breakthrough events [1]. In an acute-seizure cohort, objective therapeutic drug monitoring identified nonadherence in 39% of admissions, including patients who reported regular intake [11].	Among proposed precipitants, nonadherence has one of the clearest mechanistic and objective relationships to reduced antiseizure protection.	Breakthrough-specific trigger attribution was retrospective and was not systematically verified using therapeutic drug monitoring; objective adherence evidence derives largely from acute seizure admissions rather than narrowly defined breakthrough cohorts.
Reduced effective ASM exposure and medication-related factors	Bauer and Quigg [12]	Changes in therapy, concurrent medications that alter ASM pharmacokinetics, and inadvertent exposure to proconvulsant medications may contribute to transient loss of seizure control in medically responsive epilepsy.	Loss of seizure control may reflect reduced pharmacologic protection or a lowered seizure threshold despite reported adherence.	Evidence is derived largely from broader literature on medically responsive epilepsy rather than prospective cohorts meeting the narrower breakthrough-seizure definition used in this review.
Sleep deprivation	Doerrfuss et al. [1]; Rossi et al. [13]; Samsonsen et al. [14]	Sleep deprivation was documented in 5.3% of breakthrough events [1]. A critical review found causal evidence low quality and conflicting [13], whereas a prospective case-crossover study found lower sleep time before seizure admissions than during seizure-free follow-up [14].	Biologically plausible and supported by some prospective observations, but independent causality for breakthrough seizures remains uncertain.	Most studies are not breakthrough-specific, and sleep loss commonly co-occurs with alcohol use and other exposures.
Psychological stress	Doerrfuss et al. [1]; Baldin et al. [15]; Catalán-Aguilar et al. [16]	Excessive stress was documented in 4.6% of breakthrough events [1]. Lifetime anxiety/mood variables and low collective efficacy were associated with recurrence [15], while a systematic review found frequent but heterogeneous and sometimes conflicting associations with perceived stress [16].	Stress-related factors are clinically meaningful, but trait-level vulnerability and acute proximal stress should not be treated as equivalent.	Stress is subjective, variably measured, and strongly confounded by mood symptoms, sleep, alcohol use, and adherence.
Infection or systemic illness	Doerrfuss et al. [1]	Acute infection was documented as a presumed precipitant in 5.3% of breakthrough seizures.	Illness may contribute directly or through fever, dehydration, sleep disruption, reduced intake, altered absorption, metabolic changes, or missed ASM doses.	Breakthrough-specific evidence is descriptive and lacks matched seizure-free comparison periods, making the independent effect of infection difficult to isolate.
Alcohol exposure	Hamerle et al. [18]; Samsonsen et al. [19]	Alcohol-related worsening was reported after relatively large intake (at least seven standard drinks), with most events occurring within 12 hours after cessation [18]. A prospective crossover study did not find greater modest social drinking before seizures, but binge drinking and withdrawal were associated with loss of seizure control [19].	Risk appears concentrated around heavy intake, binge drinking, and withdrawal rather than modest alcohol consumption.	Alcohol exposure often clusters with sleep loss and medication nonadherence; some evidence relies on patient attribution.
Pregnancy and altered ASM exposure	Doerrfuss et al. [1]; Pennell et al. (MONEAD) [20,21]	Pregnancy was retrospectively identified as a presumed precipitant in 3.3% of breakthrough events [1]. Dose-normalized concentrations declined for several ASMs during pregnancy [20], but prospective MONEAD data found seizure-frequency worsening in 23% of pregnant women versus 25% of nonpregnant controls [21].	Pregnancy is associated with medication-specific pharmacokinetic changes but should not be treated as a uniform independent precipitant of breakthrough seizures.	Pharmacokinetic change does not itself establish clinical seizure causation, and MONEAD was not restricted to patients meeting the ≥12-month breakthrough definition.
Clustering of precipitants	Samsonsen et al. [22]	In 152 acute seizure admissions, sleep loss, hazardous drinking, anxiety, depression, and nonadherence frequently overlapped; patients sometimes under-recognized objectively identified nonadherence or harmful drinking.	Single-trigger attribution may be artificial; multiple exposures can form a cascade during a period of elevated susceptibility.	The cohort consisted of acute seizure admissions rather than patients selected for sustained pre-event seizure freedom.

Clinical implications

The distinction between background susceptibility and proximal precipitants has practical implications for counseling and management. Background factors such as prior seizure burden, neurological impairment, treatment history, and epilepsy etiology may help inform prognosis, but should not be used as deterministic rules for decisions such as ASM withdrawal [1,2,7].

An apparent breakthrough seizure should prompt a structured reassessment rather than be dismissed as an isolated event. Evaluation should first confirm that the event was epileptic and determine whether it remains within the intended breakthrough category rather than representing an acute symptomatic seizure [6]. Clinicians should then review medication adherence, recent ASM changes, and interacting or proconvulsant medications [11,12]; sleep disruption [13,14]; alcohol or other substance exposure [6,18,19]; systemic illness [1]; and pregnancy when applicable [20,21]. The evaluation should also reconsider whether the event reflects greater underlying epilepsy susceptibility [1,2]. Adherence deserves particular attention because patient report may not reliably reflect actual medication exposure, although drug concentrations should also be interpreted in clinical context rather than as definitive proof of adherence or nonadherence [11].

Counseling about precipitants should reflect evidentiary uncertainty. Consistent sleep, medication adherence, avoidance of heavy or binge alcohol exposure, and appropriate management of illness remain reasonable recommendations, but patients should not be told that every episode of stress or sleep loss has been proven to independently cause seizures. Pregnancy similarly requires attention to changes in ASM pharmacokinetics and medication exposure rather than being viewed simply as a seizure trigger [20,21].

Overall, evaluation should integrate background susceptibility, changes in effective ASM exposure, and clusters of proximal precipitants rather than searching for a single cause.

Causality, evidence limitations, and future directions

A central limitation of the seizure-precipitant literature is the frequent transition from temporal association to causal language. Ideally, a causal precipitant should precede the seizure, occur more frequently or with greater intensity before seizures than during comparable seizure-free periods, remain associated after relevant confounders are considered, and demonstrate reproducibility within or across individuals.

Much of the breakthrough-specific evidence does not yet meet these standards. Potential precipitants have often been identified retrospectively after a breakthrough seizure rather than systematically measured during both seizure and seizure-free periods [1]. This approach is vulnerable to recall bias, as patients may search for an explanation after an unexpected seizure, and ascertainment bias, because the frequency of the same exposure during periods without seizures is often unknown. Causal attribution is further complicated by the clustering of sleep deprivation, alcohol use, psychological distress, medication nonadherence, illness, and other exposures [14,19,22].

Sleep deprivation and psychological stress illustrate these limitations particularly well. Both are commonly reported and biologically plausible, yet the evidence supporting an independent causal relationship remains inconsistent or methodologically limited [13-17]. For many proposed precipitants, the most appropriate interpretation is therefore not “proven cause,” but rather commonly reported, biologically or clinically plausible, and incompletely established as an independent causal factor.

Addressing these limitations will require more rigorous breakthrough-specific research. A standardized definition should specify the required duration of seizure freedom, continued ASM treatment, recent medication changes, and the classification of seizures occurring with nonadherence. Prospective studies should measure candidate precipitants both before seizures and during seizure-free control periods, with within-patient case-crossover designs offering a particularly useful approach for reducing confounding by stable patient characteristics.

Objective measurements should also replace retrospective self-report when feasible. Drug concentrations or electronic medication monitoring could improve assessment of adherence, while wearable devices and sleep tracking may better characterize sleep exposure. Repeated standardized measures could similarly improve assessment of stress and alcohol use. Background risk-factor and etiologic associations identified in existing cohorts also require validation in larger, multicenter populations.

Finally, future studies should examine external precipitants alongside fluctuations in endogenous seizure susceptibility. Integrating behavioral, pharmacological, physiological, and electrophysiological data may help determine not only which exposures are associated with breakthrough seizures, but when and in whom those exposures are most likely to matter [23,24].

## Conclusions

Breakthrough seizures are best understood as the result of an interaction between underlying epilepsy susceptibility and proximal exposures rather than a single universal trigger. Background factors such as seizure burden, difficulty achieving remission, neurological impairment, treatment history, and epilepsy etiology appear to influence baseline risk, while evidence for many commonly cited precipitants remains less certain.

The central clinical message is to distinguish what makes a patient vulnerable from what may precipitate an individual seizure. Medication nonadherence or other reductions in effective ASM exposure, heavy alcohol exposure, altered ASM exposure during pregnancy, sleep loss, stress, and illness may contribute, often in combination, but should not automatically be assumed to be causal. This framework supports more accurate counseling, targeted evaluation of reversible factors, and future research designed to distinguish association from true causation.
